# The Intracellular Citrus Huanglongbing Bacterium, ‘*Candidatus* Liberibacter asiaticus’ Encodes Two Novel Autotransporters

**DOI:** 10.1371/journal.pone.0068921

**Published:** 2013-07-11

**Authors:** Guixia Hao, Michael Boyle, Lijuan Zhou, Yongping Duan

**Affiliations:** 1 United States Horticultural Research Laboratory, United States Department of Agriculture-Agriculture Research Service, Fort Pierce, Florida, United States of America; 2 Smithsonian Marine Station, Fort Pierce, Florida, United States of America; University of Würzburg, Germany

## Abstract

Proteins secreted by the type V secretion system (T5SS), known as autotransporters, are large extracellular virulence proteins localized to the bacterial poles. In this study, we characterized two novel autotransporter proteins of ‘ *Candidatus* Liberibacter asiaticus’ (Las), and redesignated them as LasA_I_ and LasA_II_ in lieu of the previous names Hyv_I_ and Hyv_II_. As a phloem-limited, intracellular bacterial pathogen, Las has a significantly reduced genome and causes huanglongbing (HLB), a devastating disease of citrus worldwide. Bioinformatic analyses revealed that LasA_I_ and LasA_II_ share the structural features of an autotransporter family containing large repeats of a passenger domain and a unique C-terminal translocator domain. When fused to the GFP gene and expressed in *E. coli*, the LasA_I_ C-terminus and the full length LasA_II_ were localized to the bacterial poles, similar to other members of autotransporter family. Despite the absence of a typical signal peptide, LasA_I_ was found to localize at the cell surface by immuno-dot blot using a monoclonal antibody against the partial LasA_I_ protein. Its surface localization was also confirmed by the removal of the LasA_I_ antigen using a proteinase K treatment of the intact bacterial cells. When co-inoculated with a P19 gene silencing suppressor and transiently expressed in tobacco leaves, the GFP-LasA_I_ translocator targeted to the mitochondria. This is the first report that Las encodes novel autotransporters that target to mitochondria when expressed in the plants. These findings may lead to a better understanding of the pathogenesis of this intracellular bacterium.

## Introduction

Autotransporters are large multi-domain virulence factors encoded by genomes of diverse gram-negative bacteria. A typical autotransporter consists of three functional domains: a Sec-dependent N-terminal signal peptide, a secreted passenger domain (α-domain) and a conserved C-terminal translocator domain (β-domain) [1]. The central passenger domain will ultimately be either attached to the cell surface or secreted. This type of self-transporting protein system is referred to as a type V secretion system (T5SS). Known virulence factors secreted by T5SS have been shown to be cytotoxic, contain protease activities, or functions such as adhesions. Based on structural features, autotransporters have recently been classified into three sub-types: classical autotransporters (T5aSS), two-partner secretion system (T5bSS) and trimeric autotransporters (T5cSS) [2]. The signal peptide directs export of the precursor protein across the inner membrane using the Sec machinery and then is cleaved by peptidase. Subsequently, the β-domain inserts into the outer membrane and forms a pore with 12 transmembrane β-strands through which the passenger domain is presumed to be exported [3]. Once the passenger domain is translocated to the cell surface, it is usually cleaved from the translocator domain and released extracellularly. In some cases the passenger domain is not cleaved and remains tightly associated with the cells [4]. The trimeric autotransporters (T5cSS) known as AT-2 are exemplified by the oligomeric coiled-coil adhesions from various pathogenic bacteria, such as YadA of *Yersinia* [5], Hia of *Haemophilus* [6], and Hap of *Haemophilus* [7]. Compared with the conventional translocator domain that typically contains about 300 amino acids, AT-2 contains a short translocator domain of about 70 amino acids that is sufficient for translocation of the passenger domains [8,9]. Deletion of the YadA translocator domain abolishes the ability to insert into the outer membrane [9]. Many AT-2 passenger domains contain large repeat units of about 70 residues. Phylogenetic clustering of these repeat units revealed that they share striking clustering patterns in which some of the repeats are almost identical in sequence [10]. It has been reported that autotransporters from a variety of rod-shaped pathogenic bacteria, including IcsA and SepA of *Shigella flexneri*, AIDA-I of *Escherichia coli*, and BrkA of *Bordetella pertussis*, are localized to the bacterial poles [11]. Recently, it was demonstrated that the YadA translocator localized solely to the mitochondrial outer membrane when expressed in yeast and that four β-stands are sufficient for mitochondrial localization [12].

‘ *Candidatus* Liberibacter asiaticus’ is a Gram-negative, fastidious alpha-Proteobacterium, causing huanglongbing (HLB), a devastating disease of citrus worldwide. HLB causes rapid decline and shortens the life span of infected trees [13]. Having a greatly reduced genome of approximately 1.23 Mb, Las bacteria reside in phloem sieve cells of infected citrus plants and are transmitted by the citrus psyllids, *Diaphorina* *citri* [14,15]. Intriguingly, even with such a small genome size, the Las psy62 genome contains multiple prophage-related regions, and two were identified as prophages/temperate phages, which occupy ca. one-sixteenth of the entire Las genome [15,16]. Within these prophage regions, two hypothetical hypervariable proteins (Hyv_I_ and Hyv_II_) were identified that contained multiple, nearly-identical, leucine-rich repeats (LRRs). The diversity and plasticity of these two genes may have implications for how these intracellular bacteria adapt to their host ecological niches [17].

Prophages in many bacterial genomes are associated with bacterial pathogenicity and biofilm formation [18]. In the present study, we discovered that these two hypervariable proteins encoded by Las prophages are novel autotransporters (redesignated as LasA_I_ and LasA_II_). We determined that LasA_I_ and LasA_II_ are polar and surface localized in bacteria in addition to being targeted to the mitochondria when expressed in plant cells. Previously, we demonstrated that the Las bacterium may act as an “energy parasite” by encoding a functional ATP translocase for direct ATP/ADP importation from their host cells [19]. Together these findings may lead us to understand how these intracellular bacteria modulate their host energy biosyntheses during their pathogenesis.

## Results

### Characteristics of unique autotransporters, *lasA*_I_ and *lasA*_II_, in Las

The *lasA*_I_ and *lasA*_II_, previously reported as *hyv*_I_ and *hy*v_II_, are located in two prophage regions in the Las Psy62 genome [17]. The 2760 bp *lasA*_I_ encodes a 919 amino-acid hypothetical protein with a predicted molecular mass of 103.5 kDa. LasA_I_ contains 12 full nearly identical leucine-rich repeats (LRRs) and 4 partial LRRs. The repeated sequence of the motifs is LEQIDLSKLEQIDLSEMAVLTQKMNIIDGIVNNLATQTKDVGRK.  The 12 full repeats within the *lasA*_I_ gene share 93-100% identity at the nucleic acid level and 84-100% identity at the protein level. *lasA*_II_ contains 1026 bp and encodes a 341 amino-acid hypothetical protein with a predicted molecular mass of 38.9 kDa. LasA_II_ has only one partial LRR. The translocator domains of LasA_I_ and LasA_II_ share 80% identity at the amino acid level while the passenger domains share 50% identity at the amino acid level. Using the SignalP signal peptide prediction software, no signal sequence was identified in LasA_I_ or LasA_II_ and little information about the function of LasA_I_ or LasA_II_ was obtained from a BLAST search of the NCBI protein database. The LasA_I_ and LasA_II_ passenger domains share low level (about 25%) amino acid sequence similarities with the LRR protein of *Colwellia psychrerythraea* 34H (GenBank accession number: AAZ26055) and the cell wall associated biofilm protein of *Staphylococcus epidermidis* (ZP_06614153). Surprisingly, the passenger domains of LasA_I_ and LasA_II_ share similar LRR repeat structures with the Toll-like receptors (TLRs) that function as sentinels of the innate immune system by binding a variety of ligands, including lipopolysaccharide, flagellin and dsRNA, through a LRR ligand-binding domain [20]. LasA_I_ and LasA_II_ translocator domains were predicted to contain ten and twelve β-stranded secondary structures respectively by the YASPIN Secondary Structure Prediction program. However, the 3D structure predicted by the I-TASSER program did not form the typical β-barrel structure, which was reported for the translocator domain of the autotransporter NalP from *Neisseria* *meningitidis* [3,21]. Despite the absence of typical signal peptides and no significant sequence homology with other autotransporters at the amino acid level, sequence analyses predicted that LasA_I_ and LasA_II_ possess architectural features of the autotransporter family, including passenger domains with large repeated sequences that form coiled-coils and translocator domains containing β-stranded structures.

### LasA_I_ is an outer membrane protein and non-cleaved from cells

**Figure 1:**
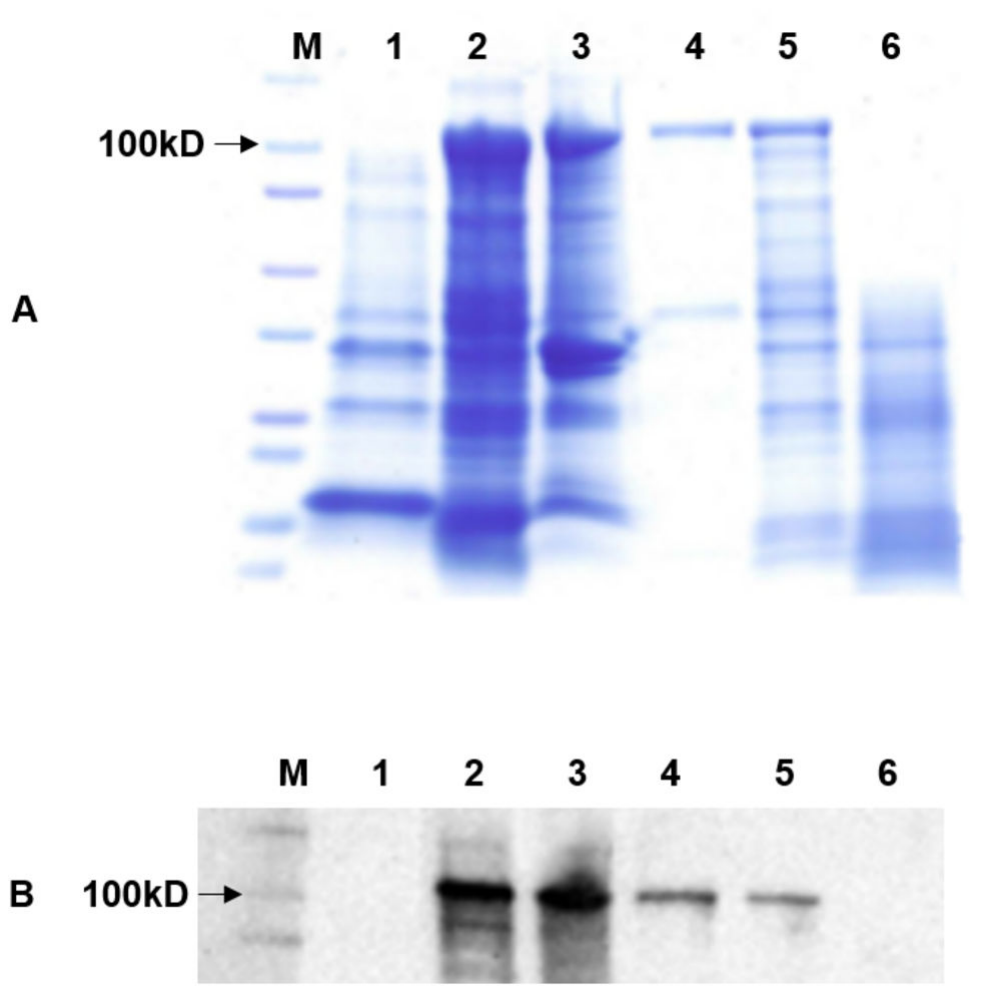
Expression and outer membrane localization of a novel autotransporter protein, LasA_I_, of ‘ *Candidatus* Liberibacter asiaticus’ (Las). SDS-PAGE (**A**) and Western blot (**B**) analysis of *E. coli* containing the pET102-*lasA*_I_ construct. M: A molecular mass marker; lane 1: Whole-cell lysate from *E. coli* BL21 containing plasmid pET102 alone; lane 2: Whole-cell lysate from *E. coli* BL21 containing recombinant plasmid pET102-*lasA*_I,_ lane 3: outer membrane protein, lane 4: purified LasA_I_ from whole-cell pellet, lane 5: LasA_I_ protein without proteinase K treatment, Lane 6: LasA_I_ with proteinase K treatment.

The full length gene *lasA*_I_ was cloned into the pET102D-TOPO vector and protein expression was induced in *E. coli* BL21 (DE) cells (Invitrogen, Carlsbad, CA). A protein of the expected size for LasA_I_ was shown on SDS-PAGE and confirmed by Western blot (Figure 1). LasA_I_ was purified under hybrid conditions, and the elution fractions contained two bands detected by SDS-PAGE. The 120 kDa protein band including the 16 kDa fusion tag was verified by Western blot with an antibody against LasA_I_ (N terminus, one full repeat and amino acids from part of the translocator domain). No signal was detected for the 40 kDa protein on the same Western blot (Figure 1B).

It has been shown that autotransporter passenger domains are transported to the cell surface and most of them are processed, thus releasing the passenger domain into the culture’s supernatant. To determine the subcellular localization of LasA_I_ and its passenger domain, outer membrane proteins, surface-associated proteins and secreted proteins in culture supernatant were isolated. As shown in Figure 1, a 120 kDa protein was detected in outer membrane fraction by SDS-PAGE gel and confirmed by western blot, which suggests LasA_I_ containing both passenger domain and translocator domain in these fractions. In contrast no LasA_I_ protein was detected from the culture supernatant using the anti-LasA_I_ antibody (Figure S1A), which suggests that even though the LasA_I_ protein contains the signal information required for cell pole localization in *E. coli*, its passenger domain was not cleaved and released into the culture’s supernatant in *E. coli*. The surface-associated protein was isolated from cell pellets and a Western blot was performed using an anti-LasA_I_ antibody. No specific binding signal was observed (Figure S1A). Taken together, the results indicate that the LasA_I_ is an outer membrane protein and its passenger domain was not cleaved and was still tightly associated with the translocator domain in *E. coli*.

### Polar localization of LasA_I_ and LasA_II_

**Figure 2:**
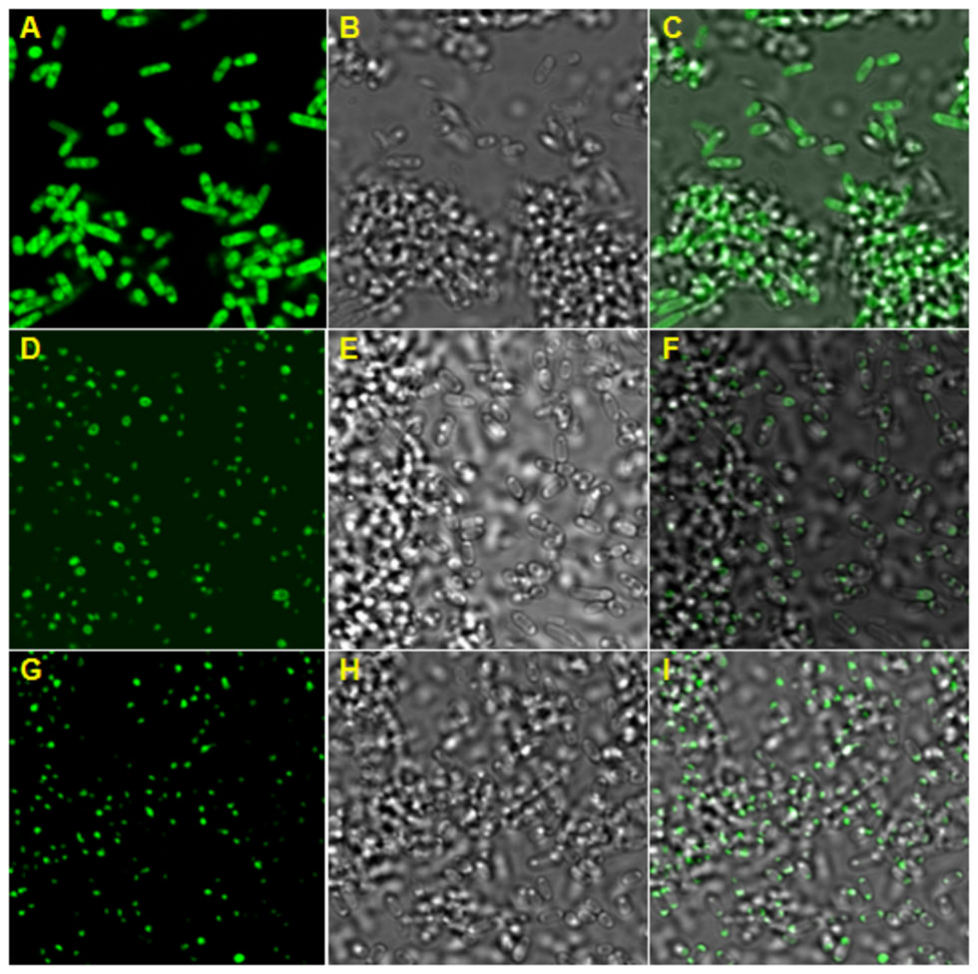
Polar localization of Las autotransporters LasA_I_ and LasA_II_ from ‘ *Candidatus* Liberibacter asiaticus’ (Las) in ***E. coli** *. **A**, **D**, **G**: GFP expression detected by confocal laser scanning microscopy (CLSM) in *E. coli* containing recombinant plasmids pET102-*gfp*, pET102-*gfp*-*lasA*_I_-TD and pET102-*gfp*-*lasA*_II_, respectively. **A**, **D**, **G**: 505 nm LP filter; **B, E, H**: differential interference contrast (DIC) of bacterial cells; **C, F, I**: FITC-DIC merged.

Several autotransporters from a variety of rod-shaped pathogenic bacteria are polar-localized in the bacterial [11]. We examined the localization of LasA_I_ and LasA_II_ by constructing GFP fusion proteins. The expression of GFP and GFP fusion proteins was detected by Western blot with an anti-GFP antibody (Figure S1B). When GFP was fused with the translocator domain of *lasA*_I_ (pET102-gfp-*lasA*_I_-TD), or the full length *lasA*_II_ gene (pET102-gfp-*lasA*_II_), the expression of GFP was observed at the cell poles of *E. coli* by confocal laser scanning microscopy (CLSM) (Figure 2D–I). In the control panel, the expression of GFP (pET102-gfp) without the fusion partner was observed in the whole cell, which indicates GFP itself is not directed to bacterial cell poles (Figure 2A–C).

### Exportation of the LasA_I_ and LasA_II_ passenger domains by the translocator domains

**Figure 3:**
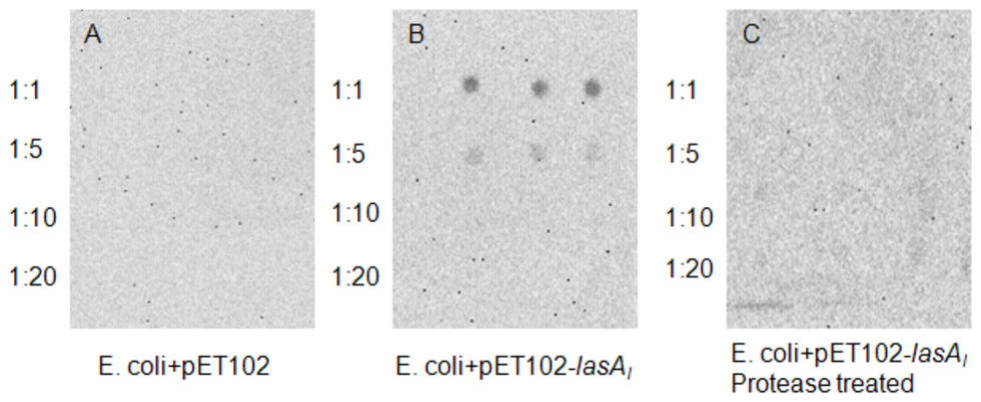
Surface location in *E. coli* of the autotransporter LasA_I_ as determined by immuno-dot blot analysis. Serial dilutions of *E. coli* cells were deposited onto a nitrocellulose membrane. The presence of LasA_I_ was detected with anti-LasA_I_ antibody. **A**: *E. coli* containing control plasmid pET102; **B**: IPTG-induced *E. coli* containing recombinant plasmid pET102-*lasA*_I_; **C**: IPTG induced *E. coli* containing recombinant plasmid pET102-*lasA*_I_ treated with Proteinase K.

Although the typical N-terminal signal sequence found in most autotransporters was not identified in LasA_I_, our results showed that the passenger domain of the LasA_I_ protein is localized at the *E. coli* cell surface. Immuno-dot blot results showed strong signals indicating LasA_I_ passenger domain is transported out of the bacterial cells when expressed in *E. coli*, and no signal was observed in the control strain of *E. coli* (Figure 3). Proteinase K-treated *E. coli* containing the LasA_I_ constructs did not bind the LasA_I_ antibody, indicating that the passenger domain of LasA_I_ was degraded on the surface of the bacterial cells. LasA_I_ degradation by proteinase K was confirmed by SDS-PAGE and Western blot. Proteinase K-treated *E. coli* cells expressing LasA_I_ contained no signal while the untreated control cells contained the full 120 kDa protein (Figure 1). Proteinase K has no ability to cross the bacterial membrane and only digest the surface protein of intact bacteria. This confirmed that the passenger domain of LasA_I_ was digested on the surface of the bacterial cells.

**Figure 4:**
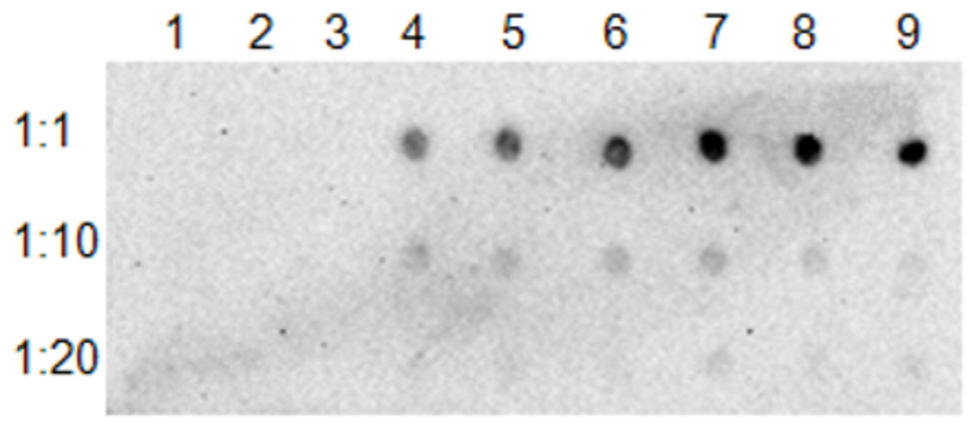
Surface localization in *E. coli* of GFP fusion proteins of LasA_I_ and LasA_II_ by immuno-dot blot. Serial dilutions of bacterial cells were deposited onto a nitrocellulose membrane; GFP was detected with anti-GFP antibody. **Lane 1, 2, 3**: IPTG induced *E. coli* containing plasmid pET102-*gfp*; **Lane 4, 5, 6**: IPTG-induced *E. coli* containing plasmid pET102-*gfp*-*lasA*_I_-TD; **Lane 7, 8, 9**: IPTG induced *E. coli* containing plasmid pET102-gfp-*lasA*_II_.

The LasA_I_ translocator domain not only exports its native passenger domain but also the LasA_I_-GFP fusion protein to the cell surface. When GFP alone was expressed, the GFP protein stayed inside the cells and no GFP binding signal was detected with an anti-GFP antibody. However, when GFP was fused with the translocator domain of *lasA*_I_, or the full length *lasA*_II_ gene, the GFP proteins were detected on intact bacterial surfaces with an anti-GFP antibody (Figure 4). After proteinase K digestion, immuno-dot blot results showed no GFP signal (data not shown). However, when the whole-cell lysate was treated with proteinase K and analyzed by SDS-PAGE, a slight reduction in the intensity of the fusion proteins was observed with Coomassie blue staining (data not shown). This indicated that not all fusion proteins expressed in *E. coli* are exposed on the surface of the bacteria and that the translocation of the GFP fusion protein is not as efficient as with the native LasA_I_ passenger domain.

To further confirm the surface localization of LasA_I_, an immunofluorescence assay (IFA) was performed. The *E. coli* cells expressing the LasA_I_ protein were not labeled by anti-LasA_I_ antibody even though the isolated LasA_I_ protein and the *E. coli* cells expressing the LasA_I_ protein can be detected by Western blot and immuno-dot blot. The absence of surface labeling indicates that the LasA_I_ protein produced in *E. coli* may not be secreted and folded properly.

### LasA_I_ targeting to mitochondria

**Figure 5:**
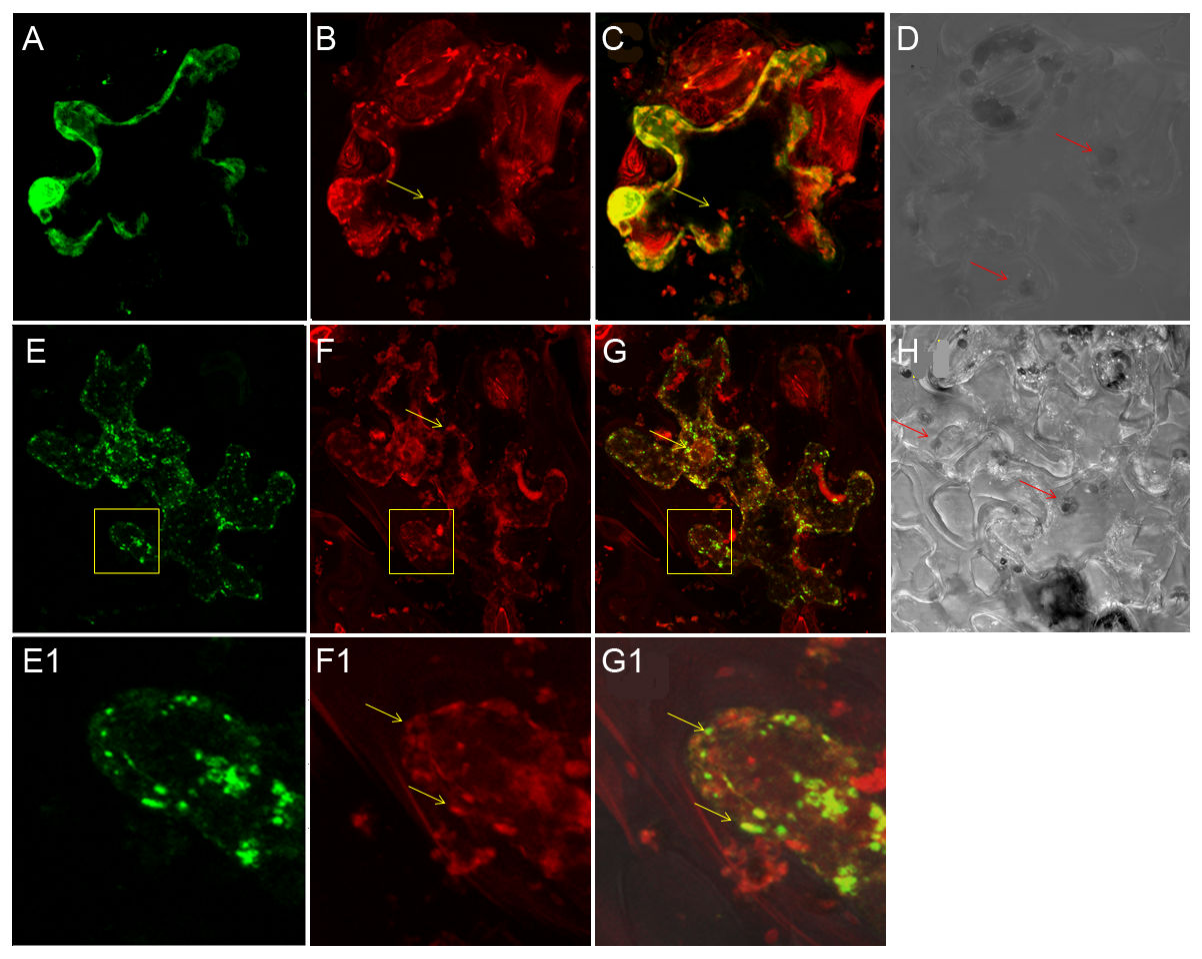
Mitochondrial localization of the Las autotransporter LasA_I_. **A-G1**: confocal laser scanning micrographs. GFP expression and MitoTracker labeling were detected in tobacco leaves infiltrated with pGDY and pGDY-*lasA*_I_-TD plasmids, respectively. **A**, **E**: GFP detection with 505-530 nm BP filter; **B**, **F**: MitoTracker detection with 560 nm LP filter; **C**, **G**: merged scans; **D**, **H**: differential interference contrast (DIC) micrographs of tobacco cells with chloroplasts (red arrows). **E1, F1, G1**: magnifications of yellow boxes in panels E, F and G. Mitochondria (yellow arrows).

**Figure 6:**
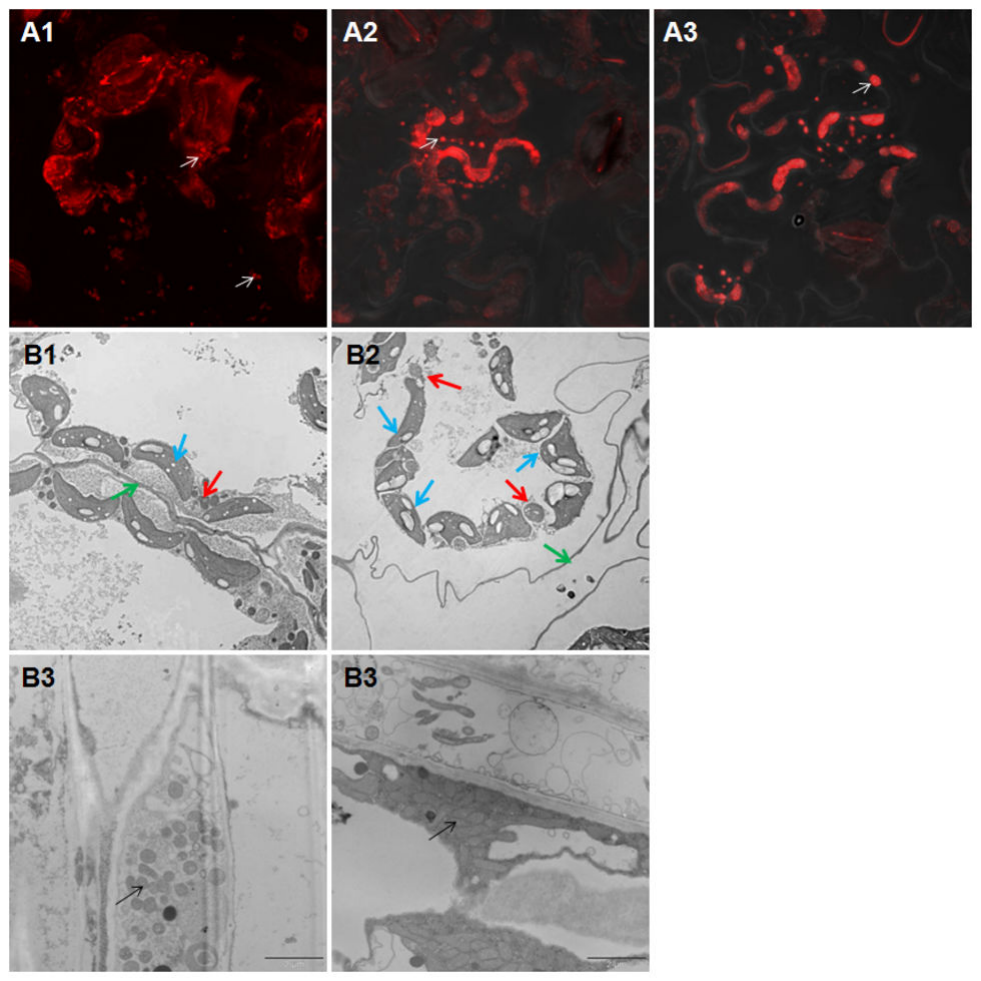
Mitochondria aggregation and morphology in plant cells. **A1-A3**: confocal laser scanning micrographs. MitoTracker labeling was detected in tobacco leaves infiltrated with pGDY (A1), pGDY-*lasA*_I_ (A2) and pGDY-*lasA*_II_ (A3). Arrows indicate normal and aggregated mitochondria respectively. **B1-B4**: transmission electron microscopy micrographs. **B1**: normal mitochondria (red arrow), chloroplast (blue arrow) and cell wall (green arrow) from infiltrated pGDY. **B2**: enlarged mitochondria (red arrow), abnormal chloroplast (blue arrow) and detached cell wall (green arrow) from infiltrated pGDY-*lasA*_I_. **B3**: normal mitochondria (arrow) from healthy periwinkle. **B4**: aggregated abnormal mitochondria (arrow) from Las-infected periwinkle.

The autotransporter YadA translocator domain was expressed in yeast and imported into the mitochondria, which did not interfere with mitochodrial function [12]. To investigate the potential function and cellular localization of LasA_I_ and LasA_II_ in plant cells and the role of the LasA_I_ and LasA_II_ translocator domains, full-length *LasA*_I_, full-length *LasA*_II_ and the translocator domain of *LasA*_I_ were cloned into the pGDY vector and transformed into *Agrobacterium tumefaciens* strain GV 2660. Transient expression results showed no detectable GFP in tobacco plants inoculated with these constructs, except with the pGDY vector alone. Co-inoculation of tobacco leaves with a P19 gene silencing suppressor and pGDY-*lasA*_I_-TD construct facilitated GFP expression (data not shown). Only a few cells had detectable GFP in the infiltrated zone when the full length *lasA*_I_ or *lasA*_II_ constructs were co-inoculated with the gene silencing suppressor. CLSM and propidium staining results showed that the expression of pGDY-*lasA*_I_-TD appeared to localize in the mitochondria. MitoTracker labeling and CLSM confirmed that GFP-*lasA*_I_-TD targeted to the mitochondria in tobacco leaves. As shown in Figure 5G and G1, the appearance of yellow mitochondria confirmed the localization of the pGDY-*lasA*_I_-TD fusion protein. The autofluorescence of chloroplasts was not observed by MitoTracker detection using a 560nm low-pass filter as shown by differential interference contrast (DIC) (Figure 5 D and H). In contrast, GFP alone in whole cells expressed mainly in the nucleus and did not show yellow mitochondria (Figure 5C). Our results demonstrated that the translocator domain of LasA_I_ contains sufficient structural information for targeting mitochondria. No obvious cell death response was observed in the infiltrated leaf zone; however, mitochondria aggregation was observed when infiltrated with full length *lasA*_I_ and *lasA*_II_ constructs (Figure 6A2 and A3). In infiltrated leaves, enlarged mitochondria and morphology change in chloroplast were observed and both of them are detached from cell wall (Figure 6B2). In addition, aggregation and changes in mitochondrial morphology were observed in infected periwinkle (Figure 6C2). Collectively, these results suggest that *lasA*_I_ and *lasA*_II_ may affect mitochondria and chloroplast function and manipulate energy production during Las infection.

## Discussion

We previously reported the genetic diversity and characteristics of two hypervariable proteins (HyvI and HyvII) from the Psy62 Las genome and global Las isolates that contain up to 12 nearly identical tandem repeats [17]. In the present study, we discovered that LasA_I_ and LasA_II_ are two novel autotransporters. Most known autotransporters are virulence proteins in animal and human pathogens [2]. Typically autotransporters contain an N-terminal signal peptide, a passenger domain and a C-terminal translocator domain. However, no typical signal peptide was predicted in LasA_I_ or LasA_II_, and while the amino acid sequences of LasA_I_ and LasA_II_ translocator domains contained predicted β-stranded structures, they shared no homology with translocator domains from other autotransporters. We propose that these proteins are new members of the autotransporter family because the translocator domains of LasA_I_ and LasA_II_ not only deliver their native passenger domains, but also exported GFP fusion proteins (GFP-LasA_I_-TD and GFP-LasA_II_) onto the bacterial cell surface. These findings reveal that LasA_I_ and LasA_II_ are unique autotransporters and the T5SS may play an important role in Las pathogenesis. Furthermore, using transient gene expression in tobacco leaves, we demonstrated that LasA_I_ contains sufficient structural information for targeting the host mitochondria as do other members of the autotransporter family [12].

Secreted proteins play a central role in the interactions of bacteria and their hosts. Gram-negative bacteria have evolved several specialized secretion systems to deliver effectors into their hosts, such as the Type Ш secretion system (T3SS) and the Type IV secretion system (T4SS). As an intracellular bacterium, Las does not have a T3SS or a T4SS, but may use the Sec secretion system [15,22]. Among the known bacterial secretion systems, the autotransporter or T5SS is the simplest pathway. Since the first report in the 1980s, the autotransporter family has been continuously expanding. Most of the characterized T5SS secreted proteins contribute to the virulence of animal or human pathogens [2]. The relatively few autotransporters reported from plant pathogens include the adhesins HecA/HecB of *Erwinia* *chrysanthemii* [23] and the XatA of *Xylella fastidiosa* which is important for virulence and is also associated with bacterial autoaggregation and biofilm formation [24]. In addition, the EstA autotransporter was reported as a member of the esterase family from the rice root colonizing and beneficial bacterium, *Pseudomonas* *stutzeri* A15 [25].

The amino acid sequences of autotransporters are highly divergent except for the conserved translocator domain. LasA_I_ and LasA_II_ proteins are unique autotransporters because they share no homology with any other members of the autotransporter family. Only coiled-coil domains were predicted and no signal peptides or anchor domains were identified in the LasA_I_ and LasA_II_ proteins when using the Trimeric Autotransporter Adhesins (TAAs) domain annotation tool [26]. Since the translocator domains of LasA_I_ and LasA_II_ have the ability to export their native passenger domains and GFP fusion proteins to the *E. coli* cell surface, the C-terminal translocator domains of LasA_I_ and LasA_II_ may form β-barrel structures through which the passenger domain can pass [2]. However, using the I-TASSER program to predict the 3D structures of LasA_I_ and LasA_II_, the β-barrel structures of these translocator domains are atypical [21]. In other translocator domains, such as that of NalP from *Neisseria* *meningtidis* , the crystal structure contains a 12-stranded β-barrel with a hydrophilic pore filled by an N-terminal α-helix [3]. It is not surprising that the predicted structures of the LasA_I_ and LasA_II_ translocator domains are different from other translocators as there are no conserved amino acids between the Las translocator domains and the known translocator domains. The crystallized structures of LasA_I_ and LasA_II_ will be important for understanding the passenger domain export mechanism.

LasA_I_ and LasA_II_ localize at bacterial poles as do other reported autotransporter members, including IcsA and SepA of *Shigella flexneri*, AIDA-I of diffusely adherent *E. coli* and BrkA of *Bordetella pertussis* [11]. It has been shown that NalP from spherically shaped *N. meningitides* and BrkA from *B. pertussis* localize at the pole of *E. coli*, suggesting that autotransporters contain information required for polar localization [11]. It is interesting to note that in the IcsA protein of *S. flexneri* two regions within the passenger domain were involved in pole targeting [27]. In contrast, the LasA_I_ translocator domain alone has the ability to target the bacterial poles. Further investigation should identify the region(s) essential for LasA_I_ and LasA_II_ to localize to the bacterial poles.

Typically T5aSS autotransporters exposed at the cell surface are proteolytically cleaved at the junction of the passenger domain and the outer-membrane embedded translocation domain [2]. Although LasA_I_ and LasA_II_ were surface-localized autotransporters, LasA_I_ was present in greater amounts in whole-cell lysates and outer membrane but undetectable in the culture supernatant, indicating either that itis inefficient cleavage in *E. coli* or that the cleaved passenger domain remains tightly associated with the translocator domain. The passenger domains of T5cSS, such as Hia from *H* *. influenza* , are usually not cleaved and stay tightly associated with the cells [4]. In *E. coli*, BrkA is proteolytically cleaved at the bacterial surface, and the extracellular domain, though cleaved, remains tightly associated with the translocator domain [11]. Further efforts to confirm the LasA_I_ and LasA_II_ surface localization failed by IFA, although production of LasA_I_ and GFP fusion proteins in *E. coli* was confirmed by immuno-dot blot and Western blot. The exported native passenger domains could not bind the primary antibody, indicating that they may not fold properly on the cell surface of *E. coli*. This was also observed with the IcsA of *S. flexneri*, which can be labeled at the surface of wild type *S. flexneri* but cannot be labeled in the *E. coli* cells expressing *IcsA* [28]. Once a pure Las culture is obtained, it will be possible to confirm the LasA_I_ and LasA_II_ surface localization by IFA and determine whether passenger domains are cleaved.

To understand the function of these novel autotransporters, *lasA*_I_ and *lasA*_II_ were constructed for *Agrobacterium* -mediated transient expression. Because no GFP expression was detected with the infiltrations containing our constructs, we used the gene silencing suppressor p19 to enhance the ectopic expression in plant leaves since post-transcriptional gene silencing (PTGS) is reported as a general feature in *Agrobacterium* -mediated transient expression [29–31]. As expected, strong GFP expression was observed when the *lasA*_I_ translocater domain (pGDY-*lasA*_I_-TD) was co-infiltrated with the p19 construct; while no GFP was detected when infiltrated with the *lasA*_I_ translocater domain (pGDY-*lasA*_I_-TD) alone. However, only a few GFP expressing cells were detected from the leaves co-inoculated with p19 and the full gene constructs of either *lasA*_I_ or *lasA*_II_ (pGDY-*lasA*_I_ and pGDY-*lasA*_II_, respectively). This result may be due to GFP-LasA_I_ and GFP-LasA_II_ fusion proteins improperly folded in the plant cells. Surprisingly, we observed the mitochondria aggregation in these infiltrated cells even though there was no detectable GFP expression. We speculate that LasA_I_ and LasA_II_ may have the ability to self-cleave and produce functional subunits targeted to the mitochondria similar to the autotransporter VacA from *Helicobacter pylori* [32]. Further investigations are underway to confirm this hypothesis. Collectively, these results suggest *lasA*_I_ and *lasA*_II_ from a Las prophage/phage (bacterial virus) can act as inducers of PTGS in plant cells. To the best of our knowledge, this is the first evidence of bacterial prophage/phage gene inducing PTGS in plants. Further characterization of the PTGS conferred by LasA_I_ and LasA_II_ may shed light on the evolution and adaptation of the Las bacterium.

With the aid of the gene silencing suppressor p19, we revealed that the LasA_I_ translocator domain-GFP fusion protein targeted to the mitochondria of tobacco leaf cells. Using the YASPIN secondary structure program, LasA_I_ and LasA_II_ were predicted to contain at least ten β-stranded structures in the translocator domain. It is worth noting that four β-strands in the YadA autotranspoter of *Yersinia* are sufficient for its mitochondrial localization in yeast [12]. Furthermore, several bacterial proteins without typical N-terminal signal sequences also target mitochondria [33], indicating that the lack of signal peptides in LasA_I_ and LasA_II_ is not exceptional.

Proteins containing tandem repeats are associated with diverse functions, and the variable numbers of tandem repeats affect the pathogenicity or antigenicity of several human and animal pathogens [34]. Deletions or insertions of these repeats within the *lasA*_I_ and *lasA*_II_ genes were reported in samples of distinct geographical origins and a single origin [17]. It is interesting that the tandem repeats of the LasA_I_ and LasA_II_ passenger domains contain characteristics of the LRR family of proteins. The LRR proteins are important for immune responses, adhesion, invasion, signal transduction, and DNA/RNA processing [35]. The LRR motif of these proteins forms a “horseshoe-shaped” molecule that provides a versatile scaffold for protein–protein interactions [36]. Several LRR proteins have been shown to be located on the cell surface and play a role in surface adherence and aggregation [37]. Compared to the tobacco cells expressing the GFP-LasA_I_ translocator domain, which did not affect mitochondrial morphology, we observed mitochondrial aggregation in cells infiltrated with full length *lasA*_I_ or *lasA*_II_. This phenomenon could be explained if the translocator domain was integrated into the mitochondrial outer membrane with the LRR passenger domain facing the cytosol, thus causing mitochondrial aggregation. By transmission electron microscopy, mitochondrial aggregation was observed in Las infected periwinkle, which agrees with our observation that mitochondrial aggregation is caused by LasA_I_ and LasA_II_ expression in tobacco. Most of the reported LRR proteins contain an N-terminal signal peptide for secretion across the bacterial membrane and a C-terminal membrane attachment region followed by a hydrophobic transmembrane [37]. In contrast, LasA_I_ and LasA_II_ lack the classical signal sequence but the translocator domain can export the LRR passenger domain across bacterial membranes. AdpC, a LRR protein lacking a signal peptide, was also reported to be located on the outer membrane surface when it was expressed in a heterologous *E. coli* host [38]. Further investigation into whether LasA_I_ and LasA_II_ passenger domains target mitochondria are critical to understanding the functions of these proteins.

In conclusion, ‘*Ca*. Liberibacter asiaticus’ is an obligate, intracellular bacterium with a significantly reduced genome. We are the first to demonstrate that Las encodes two novel autotransporters (LasA_I_ and LasA_II_) that target mitochondria when expressed in plant cells. Although the functions of these effectors remain to be elucidated, we hypothesize that Las encodes these autotransporters to modulate energy biosynthesis since Las may directly import ATP/ADP from the cytosol of host cell for its energy and biosynthesis [19]. On the other hand, these proteins may serve as suppressors for plant immune responses since Las encodes a functional flagellin that induces PAMP-triggered immunity in tobacco leaves [39]. Future work will focus on the functional elucidation of LasA_I_ and LasA_II_, including an investigation into whether LasA_I_ and LasA_II_ passenger domains target mitochondria, identification of the eukaryotic binding partners, and characterization of protein structures. These studies will lead to a better understanding of Las pathogenesis, and thereby yield a better control strategy for HLB.

## Materials and Methods

### Bacterial strains, plants and cultivation

Strains and plasmids used in this study are listed in Table 1. *Escherichia coli* Top10 (Invitrogen, Carlsbad, CA) was used as a host for plasmid construction and *E. coli* BL21 (DE3) cells (Invitrogen, Carlsbad, CA) for recombinant protein expression. *E. coli* was grown in Luria-Bertani (LB) medium at 37°C. *Agrobacterium tumefaciens* strain GV2660 was cultured at 28°C in LB and used to mediate transient expression in the leaves of *Nicotiana* *benthamiana* . Antibiotics were used at the following concentrations: carbencilin, 50 µg/mL; kanamycin, 50 µg/mL.

**Table 1:** Strains and plasmids used in this study.

**Strains or plasmids**	**Description**	**Source**
***Escherichia coli***		
TOPO10	Chemically competent cells for cloning	Invitrogen
BL20(DE3)	Chemically competent cells for protein expression	Invitrogen
**Plasmids in *Escherichia coli***		
pCR 2.1	Cloning vector, Amp^r^, Km^r^	Invitrogen
pET102D-TOPO	Expression vector, Cm^r^	Invitrogen
pET102-*gfp*	pET102D carrying *gfp*, Cm^r^	This study
pET102-*lasA*_I_	pET102D carrying *lasA*_I,_ Cm^r^	This study
pET102-gfp-*lasA*_I_ -TD	pET102D carrying *gfp* and *lasA*_I_ translocator domain, Cm^r^	This study
pET102-gfp-*lasA*_II_	pET102D carrying *gfp* and *lasA*_II,_ Cm^r^	This study
** *Agrobacterium* *tumeficiences* **		
GV2660	Strain for transient expression in the plant	40
**Plasmids in *Agrobacterium* *tumeficiences* **		
pGDY	GFP transient expression vector, Km^r^	40
pGDY-*lasA*_I_	pGDY carrying *lasA*_I_, Km^r^	This study
pGDY-*lasA*_I_ -TD	pGDY carrying *lasA*_I_ translocator domain, Km^r^	This study
pGDY-*lasA*_II_	pGDY carrying *lasA*_II_, Km^r^	This study
p19	Gene suppressor, Km^r^	31

*N* *. benthamiana* seeds were stored at 4°C for 2 days prior to germination. Subsequently the seeds were germinated in chambers programmed for cycles of 16 h light and 8 h dark at 26°C. The seedlings were then transferred into FaFard 4P mix soil in plastic containers and grown in controlled greenhouse conditions.

### Plasmid construction

For *Agrobacterium* -mediated transient expression, the full length *lasA*_I_ gene, *lasA*_II_ gene and translocator domain (TD) of *lasA*_I_ were amplified using genomic DNA from infected plants using the primers listed in Table 2. The respective PCR products were cloned into a binary vector, pGDY, in which gene expression was under control of a CaMV 35S promoter [40], generating pGDY-*lasA*_I_, pGDY-*lasA*_II_ and pGDY-*lasA*_I_-TD. The recombinant plasmids were verified by sequencing and then transformed into *A. tumefaciens* GV2660 by electroporation.

**Table 2:** Primers used in this study.

**Primer**	**Sequence**
**Primers for transient expression**	
lasA_I_ -F	5’- GCGA G A T C A*ATTAGAAAAGTAAACATGG-3’
lasA -R	5’- ATTG C T G A G*TTAGTCATCAAAATTAATAAC-3’
lasA_II_ -F	5’- ATGAGATCTA G A T C T*GAGGACACTAGAAGG-3’
lasA_I_ -TD-F	5’- ATGA G A T C T*GAGGACACTAGAAGG-3’
**Primers for protein expression**	
GFP-pET-F	5’- CACCATGGTGAGCAAGGGCGAGGA-3’
GFP-pET-R	5’- TTATCTAGATCCGGTGGATCC-3’
lasA_I_ -pET-F	5’- CACCATGATTAGAAAAGTAAACAT-3’
lasA_I_ -pET-R	5’- ATAGTCATCAAAATTAATAACTTC-3’

* Restriction enzyme sites are in italics and underlined

For protein expression, the GFP-pET-F and GFP-pET-R primers were used for PCR amplification of either GFP alone, the N-terminal GFP fusion with the translocator domain of *lasA*_I_, or the N-terminal GFP fusion with the full length *lasA*_II_ from pGDY, pGDY-*lasA*_I_-TD and pGDY-*lasA*_II_ plasmids, respectively (Table 2). Full length *lasA*_I_ was amplified from infected plant DNA with primers lasA_I_-pET-F and lasA_I_-pET-R (Table 2). PCR products for each gene were ligated into the pET102/D-TOPO vector and transformed into *E. coli* TOP10 cells according to the manufacturer’s instructions (Invitrogen, Carlsbad, CA). Selected clones were cultivated at 37°C in LB broth for plasmid isolation and sequence verification. The clones were designated as pET102-*gfp*, pET102-*lasA*_I_, pET102-*gfp*-*lasA*_I_-TD and pET102-*gfp*-*lasA*_II_.

### Purification of LasA_I_

The pET102-*lasA*_*I*_ consensus clone was transformed into *E. coli* BL21 (DE3) expression cells (Invitrogen, Carlsbad, CA). LasA_I_ expression was induced and the protein purified using ProBond™ purification system under hybrid conditions according to the manufacturer’s instructions (Invitrogen, Carlsbad, CA). The purified protein was separated using one-dimensional sodium dodecyl sulfate-polyacrylamide gel electrophoresis (SDS-PAGE) and stained with Coomassie blue. Membrane transfer was performed by iBlot according to the manufacturer’s instructions (Invitrogen, Carlsbad, CA). Western blotting was performed with a primary antibody against purified partial LasA_I_ (N-terminus, one repeat and part of the translocator domain) from an immunized mouse (ProMab Biotechnologies, Richmond, CA). Goat anti-mouse HRP-conjugated antibody was used as the secondary antibody and detected by chemiluminescence following the manufacturer’s instructions (Life Technologies, Carlsbad, CA).

### Protein fraction preparation and Western blot

Whole-cell protein lysates, culture supernatant protein (CS), outer membrane protein (OM) and surface associated protein (AD) were prepared from *E. coli* BL21 containing plasmid pET102-*lasA*_I_ as previously described [11,41–43]. For the CS fraction, culture supernatants were filtered through 0.22 µM-pore size filters and concentrated approximately 100-fold by passage through Amicon centrifuge tubes with a molecular mass limit of 50 KDa (Millipore, Billerica, MA). The final pellet was dissolved in SDS-PAGE buffer. The outer membrane protein was isolated on the basis of sarkosyl insolubility [43]. Briefly the cells were collected and broken down by sonication. Total membrane proteins were separated by ultracentrifugation at 28000 rpm for 1hr at 4^°^C. To obtain the outer membrane protein, the pellet was suspended in 20mM Tris buffer (pH7.4) containing 0.5% sarkosyl and centrifuged again at 28000 rpm for 1hr at 4^°^C. The final pellet was dissolve in SDS-PAGE buffer. To obtain the AD fraction, the protein secreted but remaining bound to the cell surface, and the cell pellets were suspended in PBS and N-hexadecane was added. The suspensions were vortexed and centrifuged, and the liquid phase was filtered. The proteins were precipitated by acetone and dissolved in SDS-PAGE buffer. Proteins were separated on SDS-PAGE and detected by Western blot as described above.

### Immuno-dot blot and proteinase K treatment

Immuno-dot blotting was performed as described previously [39]. The protein expression from bacterial cells containing different constructs was induced as described above. Bacterial cultures were centrifuged at 2,000 g for 10 min at 4 C^°^, washed three times with PBS and adjusted to a final concentration of 0.45 as determined by measurements of the optical density at 660 nm. Three microliters of each serial dilution (1:1, 1:5, 1:10 and 1:20) was spotted onto a nitrocellulose membrane in three replicates. The membrane was air dried and the Western blot procedure was performed as described above. The proteinase K treatment of intact cells was performed in PBS with 10 mM MgCl_2_ [44] and then detected by immuno-dot blot. The proteins, either untreated or treated with proteinase K, were recovered for SDS-PAGE analysis and Western blotting.

### Transient gene expression in *N benthamiana*

The *A. tumefaciens* GV2660 strain containing different constructs was cultured over night in 2 mL LB medium with the addition of antibiotics, and the following day 50 µL of the cell cultures were inoculated into new 5 mL LB aliquots containing the same antibiotics. These cultures grew until the OD_600_ value reached approximately 1.0. The cultures were then centrifuged for 10 min at 3000 g, and re-suspended in 5 mL of Agromix (10 mM MgCl_2_, 10 mM MES, and 100 µM acetosyringone). After the suspension stood at room temperature for at least 3 h, the OD_600_ value was adjusted to different optical densities with Agromix. The P19 suppressor was also cultured and treated in the same way [31]. The final cell suspension with an OD_600_ of 1.0 was mixed with the p19 suppressor and infiltrated into 4 week-old *N* *. benthamiana* leaves with a 1 mL needleless syringe. The experiments were performed with ten independent replicates.

After two days of infiltration, the infiltrated zone was excised and the epidermal layers were peeled for mitochondrial staining with MitoTracker Red CMXRos according to the manufacturer’s protocol (Invitrogen, Carlsbad, CA) and imaged using a confocal laser scanning microscope (CLSM), Zeiss LSM 510. GFP was detected with a 505-530 nm BP filter and MitoTracker was detected with a 560 nm LP filter.

### Polar localization by confocal microscope

The *E. coli* BL21 (DE3) cells containing pET102-*gfp*, pET102-*gfp-lasA*_I_-TD and pET102-*gfp-lasA*_II_ constructs were cultured and induced as described above. The collected cells were mounted in antifade solution (Invitrogen, Carlsbad, CA) for CLSM. Images were taken with a 505 nm LP filter and 40Χ or 63Χ (oil) objectives.

### Transmission electron microscopy (TEM)

Midribs were sampled from healthy and Las-infected periwinkle. The infiltrated zone was sliced from tobacco. The samples were fixed and sectioned for TEM micrographs as described previously [44].

## Supporting Information

Figure S1 LasA1 protein fractions and GFP fusion proteins. **A**: **Western blot of *E. coli* containing the pET102-*lasA*_I_ construct**. Lane1: whole-cell lysate; lane 2: culture supernatant; lane 3: cell associated protein. **B**: **Western blot of *E. coli* containing GFP and GFP fusion proteins**. Lane 1: *E. coli* containing plasmid pET102-*gfp*; lane 2: *E. coli* containing plasmid pET102-*gfp*-*lasA*_I_-TD; lane 3: *E. coli* containing plasmid pET102-gfp-*lasA*_II_.
(TIF)
